# The Individual and Combined Effects of Multiple Factors on the Risk of Soft Tissue Non-contact Injuries in Elite Team Sport Athletes

**DOI:** 10.3389/fphys.2018.01280

**Published:** 2018-09-21

**Authors:** Alireza Esmaeili, William G. Hopkins, Andrew M. Stewart, George P. Elias, Brendan H. Lazarus, Robert J. Aughey

**Affiliations:** Institute for Health and Sport (iHeS), Victoria University, Melbourne, VIC, Australia

**Keywords:** injury prevention, athlete monitoring, training load, injury history, musculoskeletal screening, subjective wellness, professional experience

## Abstract

**Aim:** Relationships between athlete monitoring-derived variables and injury risk have been investigated predominantly in isolation. The aim of this study was to evaluate the individual and combined effects of multiple factors on the risk of soft-tissue non-contact injuries in elite team sport athletes.

**Methods:** Fifty-five elite Australian footballers were prospectively monitored over two consecutive seasons. Internal and external training load was quantified using the session rating of perceived exertion and GPS/accelerometry, respectively. Cumulative load and acute-to-chronic workload ratios were derived using rolling averages and exponentially weighted moving averages. History of injuries in the current and previous seasons was recorded along with professional experience, weekly musculoskeletal screening, and subjective wellness scores for individual athletes. Individual and combined effects of these variables on injury risk were evaluated with generalized linear mixed models.

**Results:** High cumulative loads and acute-to-chronic workload ratios were associated with increased risk of injuries. The effects for measures derived using exponentially weighted moving averages were greater than those for rolling averages. History of a recent injury, long-term experience at professional level, and substantial reductions in a selection of musculoskeletal screening and subjective wellness scores were associated with increased risk. The effects of high cumulative loads were underestimated by ~20% before adjusting for previous injuries, whereas the effects of high acute-to-chronic workload ratios were overestimated by 10–15%. Injury-prone players, identified via player identity in the mixed model, were at > 5 times higher risk of injuries compared to robust players (hazard ratio 5.4, 90% confidence limits 3.6–12) despite adjusting for training load and previous injuries. Combinations of multiple risk factors were associated with extremely large increases in risk; for example, a hazard ratio of 22 (9.7–52) was observed for the combination of high acute load, recent history of a leg injury, and a substantial reduction in the adductor squeeze test score.

**Conclusion:** On the basis of our findings with an elite team of Australian footballers, the information from athlete monitoring practices in team sports should be interpreted collectively and used as a part of the injury prevention decision-making process along with consideration of individual differences in risk.

## Introduction

Injuries can negatively affect team performance and impose substantial costs to sports clubs (Hägglund et al., 2013). Quantification of injury risk factors through athlete monitoring is now common practice in elite sports settings for the main purpose of injury prevention (Taylor et al., 2012; Akenhead and Nassis, 2016). These practices include monitoring individual training loads as well as the athletes' response to training through measures such as regular musculoskeletal screening and subjective wellness (Morgan et al., 2014; Colby et al., 2017b). Non-modifiable injury risk factors such as professional experience and history of previous injuries also affect training prescription and load modification practices (Rogalski et al., 2013; Blanch and Gabbett, 2016). Previous studies have investigated the effects of these injury risk factors predominantly in isolation; however, injury is a multifactorial process and is influenced by multiple predisposing factors as well as an inciting event (Meeuwisse, 1994a). The primary aim of this study was therefore to evaluate the individual and combined effects of multiple factors on the risk of soft tissue non-contact injuries, and possible confounding effects between the risk factors.

Rolling averages have been a popular method of deriving absolute (e.g., 4-week cumulative load) and relative (e.g., acute-to-chronic workload ratio) measures of training load over various time periods (Drew and Finch, 2016). This approach has recently been criticized for overlooking the training load pattern, and disregarding the physiological principle that the effects of a training stimulus decline over time (Menaspà, 2017a). Exponentially weighted moving averages have been proposed as a better alternative to rolling averages (Menaspà, 2017b; Williams et al., 2017b); however, little evidence exists in support of the application of exponentially weighted moving averages in evaluating the risk of injuries (Drew et al., 2017; Sampson et al., 2017). The secondary aim of this study was to compare the effects of training load measures derived using rolling averages and exponentially weighted moving averages on the risk of injuries.

High acute-to-chronic workload ratios (ACWR) have consistently been associated with increased risk of injuries (Hulin et al., 2016b; Murray et al., 2017a; Stares et al., 2017). High acute load and previous injuries are also established injury risk factors (Rogalski et al., 2013; Hulin et al., 2016b; Toohey et al., 2017). These variables can both contribute to a high ACWR through an increased value of the numerator and a decreased value of the denominator used in calculation of ACWR, respectively. It has previously been speculated that training and injury history may affect the relationship between ACWR and injury risk (Blanch and Gabbett, 2016); however, no studies to date have quantified such interactions. A further aim of this study was therefore to evaluate the extent to which the effects of high ACWR are explained by acute load and previous injuries in order to further our understanding of the application of ACWR for injury prevention purposes.

## Methods

### Participants

All the 55 elite male players who were enlisted in an Australian football club over a period of two consecutive seasons participated in this study (45 in the first season and 44 in the second; mean age ± SD; 22.9 ± 3.9 years). The study was approved by Victoria University Human Research Ethics Committee, and all participants provided written informed consent in accordance with the Declaration of Helsinki.

### Seasonal structure

Pre-season training phase started in November and continued until late March of the following year. The main focus of the pre-season training program was to develop the physical capacity, technical, and tactical skills of players in preparation for the in-season phase. The in-season phase lasted from April to early September when the primary focus was on the weekend match performance as well as recovery and preparation for the next match throughout the week.

### Injury definition and recording process

An injury was defined as any training or match related incident that resulted in a missed match during the in-season phase or ≥6 days of modified training during the pre-season phase (Colby et al., 2017a). Injuries were diagnosed and recorded by the club's head physiotherapist. The dates of injury onset and return to full training were recorded along with the injury mechanism and site. Only soft-tissue (muscle, tendon, and ligament) non-contact injuries of the lower limbs were considered for the analysis as these injuries are more likely to be preventable and influenced by the investigated variables (Gabbett, 2010). It should be noted that injury dates were assigned to sessions that made the final contribution to the injury occurrence in order to eliminate possible inconsistencies caused by delayed onset of symptoms. For example, when symptoms were reported the day after a match, the injury date was recorded as the match date.

### History of previous injuries

History of previous injuries for individual players was quantified by creating two variables on each day using the injury records of the study period as well as the season prior to the commencement of the study for existing and drafted players (same injury definition and recording process as previous part). These variables were the number of days since return to full training from any previous injuries (contact and non-contact) and from a previous leg injury (contact and non-contact) in order to account for the decaying effects of previous injuries. These variables were reset to zero upon sustaining a relevant new injury and started counting up from one on the first day of return to full training.

### Training load constructs and derived measures

Internal training load was quantified using the session rating of perceived exertion method (sRPE) for all training sessions and matches (RPE multiplied by the session duration) (Foster, 1998). External training load was monitored using global positioning system (GPS)/accelerometer units for field training sessions and matches (Optimeye S5, Catapult Innovations, Australia). Player Load^TM^, total distance covered, and high-intensity running (HIR) distance (>4.17 m.s^−1^) were extracted with the software (Sprint v5.1.3, Catapult Innovations, Australia) (Aughey, 2010; Boyd et al., 2013). These four internal and external training load constructs, which are commonly monitored in team sports, were then used to calculate several absolute and relative derived measures of training load over various time periods (Drew and Finch, 2016; Williams et al., 2017a).

Cumulative loads on each day were calculated as 7, 14, 21, and 28 day rolling averages as well as 7, 14, and 28 day smoothed loads. The smoothed load is an exponentially weighted moving average of training load, which accounts for the decaying effects of training using a decay factor λ (lambda) (Hunter, 1986; Williams et al., 2017b). The smoothed load at the end of each day is calculated as [λ × (today's training load)] + [(1 – λ) × the smoothed load at the end of yesterday]. The decay factor λ defines a time constant 1/λ representing the period that contains ~2/3 of the total weighting in calculation of the smoothed load (Esmaeili et al., 2018). Decay factors of 0.14, 0.07, and 0.036 were used to calculate the smoothed loads representing time constants of 7, 14, and 28 days, respectively. Our method of labeling the time constants (time period = 1/λ) is slightly different to the one recently suggested [λ = 2/(time period + 1) = > time period = (2 – λ)/λ] (Williams et al., 2017b). We have previously demonstrated that, using our method of labeling the time constant, the smoothed load of a given period has the highest correlation with the simple cumulative load of a similar period (Esmaeili et al., 2018). The first smoothed load at the beginning of each season was calculated by assigning the first daily load observation to the accumulated smoothed load on the first day of training (Murray et al., 2017a). Calculation of rolling averages, monotony, and strain at the beginning of each season started only after enough historical data were collected for each measure (e.g., 7 days from the date of first training session for individual players for the 7-d rolling average).

Rolling average ACWR on each day was calculated as the 7-d rolling average load divided by the 28-d rolling average load (the coupled approach) (Hulin et al., 2016b; Windt and Gabbett, 2018). Similarly, smoothed ACWR on each day was calculated as the 7-d smoothed load divided by the 28-d smoothed load (Murray et al., 2017a). Training monotony on each day was quantified as the 7-d rolling average training load divided by the standard deviation of daily loads of the past 7 days (Foster, 1998). Training strain was determined by multiplying the sum of daily loads of the past 7 days into the training monotony (Foster, 1998). The daily load of the current day was included in calculation of the derived measures of training load on each day as the data were later analyzed on a daily basis.

### Professional experience

Professional experience was defined as the number of years spent in the Australian football league (AFL) system at the end of each season and was categorized in three groups of development (1–2 years), main group (3–6 years), and veterans (7+ years) (Stares et al., 2017).

### Musculoskeletal screening

Regular musculoskeletal screening (as opposed to pre-season screening) was conducted once a week prior to the first field training session of the week, which was planned 2 or 3 days after a match (in-season) or a main training session (pre-season) (Esmaeili et al., 2018). The screening tests (one attempt) were left and right dorsiflexion lunge test (for calf flexibility/ankle range of motion), sit-and-reach test (for lower back/hamstring flexibility), and adductor squeeze test (for hip adductors' strength) at three angles of hip flexion (0, 45, and 90°). The description of the tests were as follows:

#### Sit-and-reach test

Players placed their bare feet against the sit-and-reach box and their middle fingers on top of each other. They were then asked to stretch forward as far as possible and hold the position for 1 s while keeping the knees straight. The reach distance from the tip of the middle fingers relative to the toe line was recorded (Gabbe et al., 2004).

#### Dorsiflexion lunge test

A permanent tape measure was fixed on the floor with 0 cm mark at a wall junction. Players were asked to place the big toe and heel of the testing leg beside the tape. They were then instructed to lunge forward until the knee touches the wall while keeping the heel in contact with the floor. The maximum distance from the tip of the big toe to the wall was recorded (Bennell et al., 1998).

#### Adductor squeeze test

With players in a supine position, a sphygmomanometer cuff pre-inflated to 20 mmHg was placed between the knees. Players were asked to maximally squeeze the cuff and hold for 1 s and the maximum pressure displayed on the dial was recorded. The test was conducted in three hip flexion angles of 0°, 45°, and 90°C (Malliaras et al., 2009).

### Subjective wellness

Subjective wellness was assessed using a short computer based questionnaire, which has previously been developed based on the areas of interest of sports science and conditioning staff as well as the frequently used items in the athlete monitoring literature (Gastin et al., 2013). The questionnaire was completed prior to training sessions and the items included fatigue, sleep quality, general muscle soreness, mood, and stress. Each item was rated on a scale of one (feeling as bad as possible) to ten (feeling as good as possible).

### Statistical analysis

All analyses were performed with the Statistical Analysis System (version 9.4, SAS Institute, Cary, NC). A small proportion (<6%) of daily training load observations for GPS-derived constructs (total distance and high-intensity running distance) was missing, owing to poor GPS reception. Player Load (accelerometer-derived construct) was still recorded for these sessions and was used to impute the missing GPS data with a general linear mixed model (Proc Mixed). Player Load and session duration (time on the field for matches) were the fixed effects, while player identity and date were the random effects. Separate imputations were performed for matches and training sessions.

The generalized linear mixed model (Proc Glimmix) with the complementary log-log link function was used to investigate the individual, combined, and possible confounding effects of factors affecting the risk (hazard) of lower limb soft-tissue non-contact injuries. Non-training days for individual players (daily sRPE=0) as well as the days when a player was recovering from any previous injury were removed from the analyses after contributing to calculation of the derived measures of training load. The analyses were performed in three parts.

The individual effects of each potential risk factor were investigated in the first part. The effects of training load were evaluated by splitting the derived training load measures of each phase (pre-season and in-season) in each season into four quantiles (groups with nearly equal number of observations) for each player separately (individualized thresholds) (Hulin et al., 2016b; Bartlett et al., 2017). The thresholds were not individualized for relative (rolling average ACWR and smoothed ACWR) and purely distribution-based measures (monotony), as they are calculated as ratios. This approach of devising the load levels was taken to account for differences between seasons, between season phases, and between individual players. The training load levels were subjectively labeled as low, moderate-low, moderate-high, and high. Soft tissue non-contact injuries were assigned to the four levels according to their associated derived training load measure on the day of injury. No latent period was included, as the derived measures were updated and analyzed daily. Injury hazard (risk per player per exposure day) for each load level was estimated in a model where training load, season, and season phase were the fixed effects and player identity was the random effect. Within player changes between seasons were also specified with a random effect (interaction of player identity and season) but this term had zero variance. An overdispersion factor was included in the model to allow for the proportion of injuries on any given day to be not perfectly binomially distributed. The low training load level was selected as the reference group in order to calculate the hazard ratio for each level representing the effect of training load on the risk of injuries (Hopkins et al., 2007). The only exceptions were rolling average ACWR and smoothed ACWR measures, where the moderate-high level was selected as the reference group based on previous findings (Hulin et al., 2016a; Malone et al., 2017).

The individual effects of history of any previous injuries and previous leg injuries were similarly evaluated by splitting the pool of the associated variables into four quantiles. The quantile representing the longest period since a previous relevant injury was taken as the reference group. The effects of professional experience were quantified by estimating the injury hazard for the three experience groups with the main group (3–6 years) selected as the reference in calculation of hazard ratios.

Musculoskeletal screening and wellness scores were converted into z-scores (scores with a mean of 0 and SD of 1) for each individual in each season and season phase separately. The injury status of a given player on each exposure day was associated with the latest available score, typically 0–6 days previously for musculoskeletal screening and 0–2 days previously for wellness. The injury hazards associated with z-scores ≤-1 and ≤-1.5 (representing more than 1 within-subject SD and 1.5 within-subject SD reduction in those variables) were compared to the injury hazards of the reference groups (z-scores >-1 and >-1.5 respectively). The resultant hazard ratios represented the effects of substantial reductions in musculoskeletal screening and wellness scores on the risk of injuries.

In the second part, the effects of training load were evaluated after adjusting for previous leg injuries by including history of previous leg injuries, training load, season, and season phase as the fixed effects and player identity as the random effect. History of previous leg injuries was chosen over the history of any previous injuries as it showed a larger effect in the first part of the analysis. The combined effects of high training load and a recent leg injury (the level representing the shortest time since a previous leg injury) were also estimated in the model with the combination of low training load (moderate-high for ACWR variables) and the level representing the longest time since a previous leg injury taken as the reference. The random effect (player identity) was estimated as a standard deviation in log units to evaluate individual differences in injury risk. The standard deviation was doubled to interpret its magnitude (Smith and Hopkins, 2011), representing the difference between injury-prone (1 SD above the mean) and robust (1 SD below the mean) players after accounting for training load and previous leg injuries. After back-transformation, this difference was expressed as a hazard ratio.

In the third part, variables that showed substantial associations with the risk of injuries in part one of the analysis (professional experience, sit-and-reach test, adductor squeeze tests, mood, but not general muscle soreness) were re-evaluated after adjusting for training load and previous leg injuries in models similar to those in part two of the analysis. Smoothed 7-d Player Load was taken as the representative measure of training load, since the variables of interest were speculatively more likely to be influenced by acute load. The reference levels for the adjusted effects of these variables were as explained in part one. The effects of each of these variables were also quantified by estimating the combination of the highest risk level of each of the selected variables with high acute load and a history of a recent leg injury relative to the reference groups. Two reference groups were defined. The first reference group (lowest risk scenario) was the combination of low training load, long time since a previous leg injury, and the lowest risk level identified in part one for the selected variables. The second reference group (regular scenario) was the combination of all levels excluding the highest risk level for each of the variables. The effects of rolling average ACWR and smoothed ACWR were similarly evaluated after adjusting for the associated acute load (7-d rolling average load for rolling average ACWR and 7-d smoothed load for smoothed ACWR) and previous leg injuries.

The thresholds for the smallest important hazard ratio representing increase and decrease in injury risk were 1.11 and 0.90, respectively (Hopkins, 2010). The uncertainty in all effects was expressed as 90% confidence limits, and qualitatively as chances that the true value of the effect was substantial for clear effects using the following scale: <0.5%, most unlikely; 0.5% to <5%, very unlikely; 5% to <25%, unlikely, 25% to <75%, possibly; 75% to <95%, likely; 95% to <99.5%, very likely; >99.5%, most likely. The effect was deemed unclear when both the lower confidence limit was <0.90 and the upper confidence limit was >1.11 (Hopkins et al., 2009). Results were rounded and reported to two significant digits (Hopkins et al., 2011).

## Results

Sixty-five lower limb soft tissue non-contact injuries were sustained by 33 individual athletes over the study period (first season = 28, second season = 37; pre-season = 26, in-season = 39). Mean thresholds for the four levels of derived training load measures over pre-season and in-season are summarized in Table 1. Thresholds for each level were higher during pre-season compared to the in-season.

**Table 1:** Daily mean thresholds of training load levels (quantiles) for pre-season and in-season.

**Derived measure**	**Level**	**sRPE (AU)**		**Player Load (AU)**		**Total distance (m)**		**High-intensity running distance (m)**	
		**Pre-season**	**In-season**	**Pre-season**	**In-season**	**Pre-season**	**In-season**	**Pre-season**	**In-season**
7-d rolling average	High	>450	>300	>350	>310	>3,700	>3,300	>1,100	>800
	Moderate-high	350–450	270–300	280–350	260–310	3,000–3,700	2,800–3,300	820–1,100	650–800
	Moderate-low	260–349	240–269	200–279	200–259	2,100–2,999	2,200–2,799	510–819	480–649
	Low	<260	<240	<200	<200	<2,100	<2,200	<510	<480
14-d rolling average	High	>420	>290	>330	>300	>3,500	>3,100	>1,000	>760
	Moderate-high	350–420	270–290	280–330	250–300	2,900–3,500	2,700–3,100	800–1,000	630–760
	Moderate-low	260–349	240–269	210–279	210–249	2,100–2,899	2,200–2,699	560–799	500–629
	Low	<260	<240	<210	<210	<2,100	<2,200	<560	<500
21-d rolling average	High	>400	>280	>310	>290	>3,300	>3,000	>930	>730
	Moderate-high	330–400	260–280	260–310	250–290	2,800–3,300	2,600–3,000	760–930	620–730
	Moderate-low	260–329	250–259	190–259	210–249	2,000–2,799	2,200–2,599	540–759	510–619
	Low	<260	<250	<190	<210	<2,000	<2,200	<540	<510
28-d rolling average	High	>380	>280	>300	>280	>3,200	>2,900	>880	>720
	Moderate-high	310–380	260–280	250–300	250–280	2,700–3,200	2,600–2,900	740–880	620–720
	Moderate-low	250–309	250–259	190–249	210–249	2,000–2,699	2,200–2,599	560–739	520–619
	Low	<250	<250	<190	<210	<2,000	<2,200	<560	<520
7-d smoothed	High	>460	>300	>340	>300	>3,600	>3,200	>1,100	>770
	Moderate-high	380–460	270–300	280–340	250–300	3,000–3,600	2,600–3,200	830–1,100	620–770
	Moderate-low	290–379	240–269	200–279	200–249	2,100–2,999	2,200–2,599	560–829	490–619
	Low	<290	<240	<200	<200	<2,100	<2,200	<560	<490
14-d smoothed	High	>450	>290	>320	>280	>3,400	>3,000	>1,000	>720
	Moderate-high	360–450	270–290	260–320	250–280	2,800–3,400	2,600–3,000	790–1,000	620–720
	Moderate-low	290–359	250–269	200–259	210–249	2,100–2,799	2,300–2,599	570–789	530–619
	Low	<290	<250	<200	<210	<2,100	<2,300	<570	<530
28-d smoothed	High	>460	>280	>310	>270	>3,300	>2,800	>1,000	>690
	Moderate-high	360–460	270–280	250–310	250–270	2,600–3,300	2,600–2,800	770–1,000	620–690
	Moderate-low	310–359	250–269	190–249	220–249	2,000–2,599	2,300–2,599	590–769	550–619
	Low	<310	<250	<190	<220	<2,000	<2,300	<590	<550
Rolling average ACWR	High	>1.25	>1.14	>1.53	>1.22	>1.52	>1.22	>1.50	>1.24
	Moderate-high	1.01–1.25	1.03–1.14	1.10–1.53	1.05–1.22	1.10–1.52	1.05–1.22	1.06–1.50	1.03–1.24
	Moderate-low	0.82–1.00	0.92–1.02	0.86–1.09	0.87–1.04	0.85–1.09	0.88–1.04	0.78–1.05	0.83–1.02
	Low	<0.82	<0.92	<0.86	<0.87	<0.85	<0.88	<0.78	<0.83
Smoothed ACWR	High	>1.11	>1.10	>1.49	>1.16	>1.48	>1.16	>1.43	>1.16
	Moderate-high	0.97–1.11	1.01–1.10	1.16–1.49	1.02–1.16	1.15–1.48	1.02–1.16	1.09–1.43	1.00–1.16
	Moderate-low	0.86–0.96	0.92–1.00	0.92–1.15	0.89–1.01	0.91–1.14	0.89–1.01	0.86–1.08	0.84–0.99
	Low	<0.86	<0.92	<0.92	<0.89	<0.91	<0.89	<0.86	<0.84
Monotony	High	>1.11	>0.87	>0.79	>0.74	>0.79	>0.75	>0.76	>0.70
	Moderate-high	0.95–1.11	0.78–0.87	0.73–0.79	0.66–0.74	0.73–0.79	0.67–0.75	0.66–0.76	0.61–0.70
	Moderate-low	0.80–0.94	0.71–0.77	0.59–0.72	0.58–0.65	0.59–0.72	0.58–0.66	0.57–0.65	0.54–0.60
	Low	<0.80	<0.71	<0.59	<0.58	<0.59	<0.58	<0.57	<0.54
Strain	High	>3,400	>1,800	>1,900	>1,600	>20,000	>17,000	>5,500	>3,800
	Moderate-high	2,300–3,400	1,500–1,800	1,400–1,900	1,200–1,600	15,000–20,000	13,000–17,000	3,800–5,500	2,800–3,800
	Moderate-low	1,500–2,299	1,300–1,499	930–1,399	860–1,199	9,700–14,999	9,200–12,999	2,200–3,799	1,900–2,799
	Low	<1,500	<1,300	<930	<860	<9,700	<9,200	<2,200	<1,900

*sRPE, session rating of perceived exertion; AU, arbitrary units; ACWR, acute-to-chronic workload ratio*.

### The individual effects

The individual effects of training load are summarized in Table 2. High levels of cumulative measures (rolling averages and smoothed loads), rolling average and smoothed ACWR, monotony, and strain were typically associated with substantial increases in the risk of injuries. The effects were considerably larger for smoothed cumulative and relative measures compared to similar measures derived using rolling averages. High 14-d smoothed Player Load had the largest effect on the risk of injuries (hazard ratio 3.2, 90% confidence limits 1.86–5.4) compared to other measures of cumulative load. In general, the 14-d period was associated with larger increases in the risk of injuries compared to other periods for both high rolling average and high smoothed cumulative loads. High smoothed ACWR was associated with the largest absolute risk of injury (injury hazard 0.79) compared to all other training load measures. Moderate-high level of smoothed ACWR was generally associated with substantially lower risk of injuries compared to all other smoothed ACWR levels.

**Table 2:** Individual effects of training load on the risk of injuries derived from part one of the analysis^a^.

**Derived measure**	**Level**	**sRPE**	**Player Load**	**Total distance**	**High-intensity running distance**
7-d rolling average	High	0.54%, 2.1 (1.29–3.5)^***^	0.50%, 1.34 (0.86–2.1)	0.47%, 1.27 (0.81–2.0)	0.45%, 1.49 (0.91–2.4)^**^
	Moderate-high	0.42%, 1.62 (0.96–2.7)^**^	0.28%, 0.75 (0.45–1.25)	0.32%, 0.86 (0.53–1.41)	0.39%, 1.28 (0.78–2.1)
	Moderate-low	0.30%, 1.15 (0.66–2.0)	0.36%, 0.97 (0.60–1.57)	0.34%, 0.91 (0.56–1.47)	0.36%, 1.19 (0.72–1.97)
	Low	0.26%, Reference	0.37%, Reference	0.37%, Reference	0.30%, Reference
14-d rolling average	High	0.70%, 2.1 (1.30–3.2)^***^	0.64%, 1.90 (1.21–3.0)^***^	0.67%, 1.84 (1.18–2.9)^***^	0.67%, 1.99 (1.27–3.1)^***^
	Moderate-high	0.36%, 1.06 (0.63–1.8)	0.31%, 0.91 (0.54–1.55)	0.29%, 0.80 (0.47–1.35)	0.41%, 1.20 (0.73–1.97)
	Moderate-low	**0.19%, 0.55 (0.30–1.02)**^00^	0.28%, 0.83 (0.49–1.43)	0.26%, 0.71 (0.42–1.23)	**0.17%, 0.49 (0.26–0.93)**^00^
	Low	0.34%, Reference	0.34%, Reference	0.36%, Reference	0.34%, Reference
21-d rolling average	High	0.52%, 1.60 (1.02–2.5)^**^	0.42%, 1.15 (0.72–1.83)	0.37%, 1.02 (0.63–1.65)	0.55%, 1.50 (0.96–2.3)^**^
	Moderate-high	0.24%, 0.70 (0.41–1.22)	0.43%, 1.19 (0.75–1.90)	0.50%, 1.38 (0.89–2.2)	0.33%, 0.92 (0.56–1.50)
	Moderate-low	0.42%, 1.24 (0.78–1.99)	0.33%, 0.91 (0.56–1.49)	0.31%, 0.84 (0.51–1.39)	0.31%, 0.84 (0.51–1.40)
	Low	0.34%, Reference	0.36%, Reference	0.36%, Reference	0.36%, Reference
28-d rolling average	High	0.44%, 1.14 (0.72–1.78)	0.39%, 1.08 (0.68–1.74)	0.36%, 1.08 (0.67–1.77)	0.49%, 1.57 (0.98–2.5)^**^
	Moderate-high	0.28%, 0.74 (0.45–1.22)	0.36%, 0.99 (0.61–1.59)	0.36%, 1.07 (0.65–1.74)	0.36%, 1.14 (0.69–1.88)
	Moderate-low	0.35%, 0.90 (0.56–1.45)	0.35%, 0.96 (0.60–1.55)	0.39%, 1.17 (0.73–1.88)	0.30%, 0.96 (0.57–1.62)
	Low	0.38%, Reference	0.36%, Reference	0.34%, Reference	0.31%, Reference
7-d smoothed	High	0.67%, 2.5 (1.53–4.0)^****^	0.70%, 2.8 (1.70–4.6)^****^	0.70%, 2.6 (1.58–4.1)^****^	0.70%, 2.6 (1.59–4.1)^****^
	Moderate-high	0.27%, 0.99 (0.56–1.74)	0.30%, 1.17 (0.66–2.1)	0.27%, 0.99 (0.56–1.75)	0.25%, 0.91 (0.51–1.62)
	Moderate-low	0.29%, 1.05 (0.60–1.83)	0.27%, 1.06 (0.59–1.91)	0.27%, 0.97 (0.55–1.72)	0.29%, 1.05 (0.60–1.84)
	Low	0.27%, Reference	0.25%, Reference	0.28%, Reference	0.27%, Reference
14-d smoothed	High	0.56%, 2.2 (1.34–3.7)^***^	0.65%, 3.2 (1.86–5.4)^****^	0.63%, 3.1 (1.79–5.2)^****^	0.58%, 2.8 (1.65–4.9)^****^
	Moderate-high	0.43%, 1.71 (1.01–2.9)^**^	0.25%, 1.21 (0.65–2.3)	0.29%, 1.43 (0.78–2.6)	0.43%, 2.1 (1.19–3.7)^***^
	Moderate-low	0.27%, 1.06 (0.59–1.89)	0.40%, 1.95 (1.10–3.4)^**^	0.38%, 1.84 (1.04–3.3)^**^	0.29%, 1.41 (0.77–2.6)
	Low	0.25%, Reference	0.21%, Reference	0.21%, Reference	0.21%, Reference
28-d smoothed	High	0.46%, 1.69 (1.03–2.8)^**^	0.49%, 2.7 (1.50–4.8)^***^	0.49%, 2.7 (1.49–4.8)^***^	0.58%, 2.8 (1.65–4.9)^****^
	Moderate-high	0.40%, 1.48 (0.89–2.5)	0.45%, 2.5 (1.38–4.5)^***^	0.50%, 2.7 (1.53–4.9)^***^	0.34%, 1.65 (0.92–3.0)^**^
	Moderate-low	0.35%, 1.30 (0.77–2.2)	0.38%, 2.1 (1.14–3.8)^***^	0.34%, 1.83 (0.99–3.4)^**^	0.38%, 1.84 (1.04–3.3)^**^
	Low	0.27%, Reference	0.18%, Reference	0.18%, Reference	0.21%, Reference
Rolling average ACWR	High	0.58%, 2.1 (1.32–3.3)^***^	0.57%, 2.2 (1.36–3.6)^***^	0.56%, 2.4 (1.48–3.9)^****^	0.49%, 1.37 (0.88–2.1)
	Moderate-high	0.28%, Reference	0.26%, Reference	0.23%, Reference	0.36%, Reference
	Moderate-low	0.29%, 1.05 (0.62–1.79)	0.38%, 1.47 (0.88–2.5)	0.44%, 1.85 (1.11–3.1)^**^	0.33%, 0.90 (0.55–1.47)
	Low	0.29%, 1.03 (0.61–1.75)	0.22%, 0.84 (0.47–1.52)	0.22%, 0.92 (0.51–1.67)	0.24%, 0.67 (0.39–1.14)
Smoothed ACWR	High	0.70%, 4.0 (2.4–6.7)^****^	0.79%, 6.8 (3.5–13)^****^	0.79%, 6.8 (3.6–13)^****^	0.75%, 4.6 (2.6–8.1)^****^
	Moderate-high	0.18%, Reference	0.12%, Reference	0.12%, Reference	0.16%, Reference
	Moderate-low	0.27%, 1.52 (0.83–2.8)	0.22%, 1.92 (0.90–4.0)^**^	0.25%, 2.1 (1.01–4.4)^**^	0.34%, 2.0 (1.09–3.9)^**^
	Low	0.31%, 1.75 (0.98–3.1)^**^	0.38%, 3.3 (1.64–6.7)^****^	0.36%, 3.0 (1.52–6.3)^***^	0.26%, 1.59 (0.83–3.0)
Monotony	High	0.52%, 1.53 (0.96–2.4)^**^	0.54%, 1.98 (1.21–3.2)^***^	0.54%, 2.4 (1.44–4.1)^***^	0.62%, 2.0 (1.26–3.2)^***^
	Moderate-high	0.34%, 1.01 (0.61–1.67)	0.33%, 1.21 (0.72–2.0)	0.32%, 1.44 (0.82–2.5)	0.26%, 0.86 (0.50–1.48)
	Moderate-low	0.31%, 0.92 (0.54–1.54)	0.34%, 1.23 (0.75–2.0)	0.42%, 1.91 (1.16–3.2)^***^	0.32%, 1.05 (0.64–1.73)
	Low	0.34%, Reference	0.28%, Reference	0.22%, Reference	0.31%, Reference
Strain	High	0.59%, 1.81 (1.15–2.9)^***^	0.54%, 1.89 (1.15–3.1)^***^	0.46%, 1.49 (0.91–2.5)^**^	0.55%, 1.95 (1.20–3.2)^***^
	Moderate-high	0.35%, 1.06 (0.64–1.76)	0.37%, 1.31 (0.78–2.2)	0.42%, 1.37 (0.83–2.3)	0.33%, 1.16 (0.68–1.98)
	Moderate-low	0.25%, 0.76 (0.44–1.32)	0.33%, 1.14 (0.66–1.96)	0.32%, 1.05 (0.62–1.78)	0.35%, 1.22 (0.72–2.1)
	Low	0.33%, Reference	0.29%, Reference	0.31%, Reference	0.29%, Reference

a Values are injury hazard (risk per player per exposure day), hazard ratio (with 90% confidence limits). Substantial effects are in bold. Likelihood of increased risk of injuries:^*^ possibly,

** *likely*,

*** *very likely*,

****b *most likely.Likelihood of decreased risk of injuries*:

00 *Likely.sRPE, session rating of perceived exertion; ACWR, acute-to-chronic workload ratio*.

The individual effects of previous injuries and professional experience are summarized in Table 3. A recent history of injuries was associated with a higher risk of injuries when compared to the reference level. The effect was slightly larger for recent leg injuries compared to any recent injuries despite the “recent” level covering a longer period for leg injuries (<85 days) compared to any injuries (<53 days). Players with 7+ years of professional experience were at a higher risk of injuries compared to the reference level (3–6 years).

**Table 3:** Individual effects of history of previous injuries and professional experience on the risk of injuries derived from part one of the analysis.

**Variable**	**Level**	**Injury hazard, hazard ratio (90% confidence limits)**
History of any previous injuries	<53 days	0.44%, 1.81 (0.97–3.4)^**^
	53-154 days	0.32%, 1.30 (0.67–2.5)
	155-362 days	0.39%, 1.61 (0.83–3.1)
	>362 days	0.25%, Reference
History of previous leg injuries	<85 days	0.52%, 2.1 (1.19–3.7)^***^
	85-232 days	0.37%, 1.49 (0.81–2.7)
	233-364 days	0.42%, 1.68 (0.82–3.4)
	>364 days	0.25%, Reference
Professional experience	1-2 years	0.38%, 1.26 (0.69–2.3)
	3-6 years	0.30%, Reference
	7+ years	0.55%, 1.85 (0.98–3.6)^**^

Substantial effects are in bold. Likelihood of increased risk of injuries: *possibly,

** *likely*,

*** *very likely*,

****most likely.

The individual effects of weekly musculoskeletal screening and subjective wellness scores are summarized in Table 4. Substantial reductions in the sit-and-reach test and adductor squeeze tests (but not the dorsiflexion lunge tests) were associated with higher risk of injuries. The effects were larger when the threshold for a substantial reduction was set at 1.5 SD as opposed to 1 SD. Among the subjective wellness variables, only substantial reductions (worse scores) in mood were associated with an increased risk of injuries. Unexpectedly, worse scores for general muscle soreness were associated with a lower risk of injuries. The descriptive statistics for the subjective wellness items were as follows: mean of the player means: 7.8–8.2; SD of the player means: 0.44–0.61; mean of the within-player SDs: 0.5–0.68. The descriptive statistics for the musculoskeletal screening tests were nearly identical to the values previously provided in detail (Esmaeili et al., 2018).

**Table 4:** Individual effects of musculoskeletal screening and subjective wellness scores on the risk of injuries derived from part one of the analysis.

**Musculoskeletal screening**			**Subjective wellness**		
**Screening test**	***Z*-score**	**Injury hazard, hazard ratio (90% confidence limits)**	**Wellness item**	**Z-score**	**Injury hazard, hazard ratio (90% confidence limits)**
Left dorsiflexion lunge test	<−1.5	0.13%, 0.56 (0.23–1.38)	Fatigue	<−1.5	0.33%, 0.87 (0.46–1.63)
	≥−1.5	0.24%, Reference		≥−1.5	0.38%, Reference
	<−1	0.23%, 1.00 (0.59–1.69)		<−1	0.28%, 0.71 (0.41–1.22)
	≥−1	0.23%, Reference		≥−1	0.39%, Reference
Right dorsiflexion lunge test	<−1.5	0.20%, 0.86 (0.41–1.84)	Sleep quality	<−1.5	0.46%, 1.25 (0.70–2.2)
	≥−1.5	0.23%, Reference		≥−1.5	0.37%, Reference
	<−1	0.13%, 0.54 (0.25–1.14)		<−1	0.40%, 1.08 (0.67–1.76)
	≥−1	0.25%, Reference		≥−1	0.37%, Reference
Sit-and-reach test	<−1.5	0.53%, 2.6 (1.56–4.4)****	General muscle soreness	<−1.5	0.%, - Very low^a^
	≥−1.5	0.20%, Reference		≥−1.5	0.%, Reference
	<−1	0.37%, 1.75 (1.11–2.8)^**^		<−1	**0.12%, 0.28 (0.13–0.61)**^000^
	≥-1	0.21%, Reference		≥−1	0.42%, Reference
Adductor squeeze test 0°	<−1.5	0.45%, 2.1 (1.12–3.8)^***^	Mood	<−1.5	0.68%, 1.92 (1.11–3.3)^***^
	≥−1.5	0.22%, Reference		≥−1.5	0.36%, Reference
	<−1	0.30%, 1.34 (0.86–2.1)		<−1	0.53%, 1.51 (0.94–2.4)^**^
	≥−1	0.22%, Reference		≥−1	0.35%, Reference
Adductor squeeze test 45°	<−1.5	0.38%, 1.69 (0.93–3.1)^**^	Stress	<−1.5	0.55%, 1.53 (0.85–2.8)
	≥−1.5	0.22%, Reference		≥−1.5	0.36%, Reference
	<−1	0.25%, 1.06 (0.65–1.73)		<−1	0.48%, 1.33 (0.81–2.2)
	≥−1	0.23%, Reference		≥−1	0.36%, Reference
Adductor squeeze test 90°	<−1.5	0.54%, 2.9 (1.80–4.7)^****^			
	≥−1.5	0.19%, Reference			
	<−1	0.38%, 2.1 (1.44–3.2)^****^			
	≥−1	0.18%, Reference			

a *All injuries were sustained in the reference group*. *Substantial effects are in bold*. Likelihood of increased risk of injuries:*possibly,

** *likely*,

*** *very likely*,

**** *most likely*.

*Likelihood of decreased risk of injuries*:

000 * ery likely*.

### The adjusted and combined effects

Table 5 provides a summary of the effects of training load after adjusting for previous leg injuries. The effects of high training load on the risk of injuries increased by an average of 20% after adjusting for previous leg injuries when compared to the individual (unadjusted) effects of training load (Table 2). The only notable exceptions were sRPE rolling average ACWR and sRPE smoothed ACWR, where the effects decreased by ~15 and 10%, respectively after adjusting for previous leg injuries. Table 5 also shows the effects of a combination of high training load and a recent leg injury where the increase in risk of injuries was considerably larger than the individual effects of each of these high-risk conditions.

**Table 5:** Effects of training load adjusted for history of previous leg injuries and combined with a history of a recent leg injury derived from part two of the analysis^a^.

**Derived measure**	**Level**	**sRPE**	**Player Load**	**Total distance**	**High-intensity running distance**
7-d rolling average	High	0.59%, 2.2 (1.31–3.7)^***^	0.54%, 1.70 (1.05–2.7)^**^	0.50%, 1.54 (0.94–2.5)^**^	0.48%, 1.60 (0.96–2.7)^**^
	Moderate-high	0.38%, 1.43 (0.82–2.5)^**^	0.25%, 0.79 (0.44–1.39)	0.33%, 1.01 (0.59–1.73)	0.40%, 1.35 (0.80–2.3)
	Moderate-low	0.29%, 1.09 (0.61–1.94)	0.39%, 1.22 (0.73–2.0)	0.36%, 1.13 (0.67–1.90)	0.34%, 1.15 (0.67–1.97)
	Low	0.27%, Reference	0.32%, Reference	0.32%, Reference	0.30%, Reference
	High + RLI	0.91%, 5.0 (2.3–11)^****^	0.79%, 3.6 (1.70–7.6)^****^	0.73%, 3.3 (1.56–7.2)^****^	0.71%, 3.5 (1.61–7.7)^***^
14-d rolling average	High	0.79%, 2.6 (1.61–4.3)^****^	0.68%, 2.3 (1.38–3.7)^***^	0.71%, 2.2 (1.35–3.5)^***^	0.73%, 2.4 (1.45–3.9)^***^
	Moderate-high	0.32%, 1.05 (0.59–1.87)	0.31%, 1.04 (0.59–1.82)	0.29%, 0.89 (0.50–1.56)	0.40%, 1.29 (0.75–2.23)
	Moderate-low	0.20%, 0.66 (0.35–1.25)	0.28%, 0.92 (0.52–1.64)	0.25%, 0.77 (0.43–1.37)	0.18%, 0.58 (0.30–1.12)
	Low	0.30%, Reference	0.30%, Reference	0.33%, Reference	0.31%, Reference
	High + RLI	1.27%, 6.3 (2.9–14)^****^	1.01%, 5.0 (2.3–11)^****^	1.05%, 4.8 (2.3–10)^****^	1.13%, 5.6 (2.6–12)^****^
21-d rolling average	High	0.55%, 1.82 (1.11–3.0)^**^	0.40%, 1.34 (0.79–2.3)	0.35%, 1.17 (0.68–2.00)	0.55%, 1.68 (1.03–2.7)^**^
	Moderate-high	0.26%, 0.87 (0.49–1.54)	0.48%, 1.58 (0.95–2.6)^**^	0.55%, 1.81 (1.12–3.0)^***^	0.37%, 1.11 (0.66-1.87)
	Moderate-low	0.42%, 1.39 (0.84–2.3)	0.35%, 1.17 (0.69–1.99)	0.33%, 1.08 (0.63–1.84)	0.31%, 0.92 (0.54–1.59)
	Low	0.30%, Reference	0.30%, Reference	0.30%, Reference	0.33%, Reference
	High + RLI	0.80%, 4.1 (1.88–8.9)^****^	0.89%, 3.0 (1.37–6.8)^***^	0.51%, 2.6 (1.17–5.9)^***^	0.81%, 3.9 (1.78–8.4)^****^
28-d rolling average	High	0.45%, 1.31 (0.80–2.1)	0.39%, 1.35 (0.80–2.3)	0.37%, 1.37 (0.80–2.34)	0.51%, 1.90 (1.13–3.2)^***^
	Moderate-high	0.30%, 0.88 (0.52–1.49)	0.38%, 1.31 (0.78–2.2)	0.38%, 1.43 (0.84–2.42)	0.38%, 1.41 (0.83–2.4)
	Moderate-low	0.34%, 0.98 (0.59–1.63)	0.36%, 1.25 (0.74–2.1)	0.41%, 1.52 (0.91–2.6)^**^	0.29%, 1.10 (0.62–1.92)
	Low	0.34%, Reference	0.29%, Reference	0.27%, Reference	0.27%, Reference
	High + RLI	0.67%, 2.8 (1.29–6.2)^***^	0.59%, 3.0 (1.33–6.6)^***^	0.54%, 3.0 (1.32–6.7)^***^	0.78%, 4.4 (1.95–9.7)^****^
7-d smoothed	High	0.75%, 3.1 (1.85–5.3)^****^	0.76%, 3.4 (2.0–5.9)^****^	0.77%, 3.1 (1.84–5.2)^****^	0.77%, 3.2 (1.88–5.3)^****^
	Moderate-high	0.26%, 1.06 (0.57–1.99)	0.31%, 1.37 (0.74–2.6)	0.28%, 1.13 (0.61–2.1)	0.25%, 1.03 (0.55–1.92)
	Moderate-low	0.31%, 1.29 (0.72–2.3)	0.27%, 1.21 (0.64–2.3)	0.27%, 1.09 (0.52–2.0)	0.29%, 1.19 (0.65–2.16)
	Low	0.24%, Reference	0.22%, Reference	0.25%, Reference	0.25%, Reference
	High + RLI	1.15%, 7.3 (3.3–16)^****^	1.12%, 7.9 (3.5–18)^****^	1.12%, 7.1 (3.2–16)^****^	1.15%, 7.3 (3.3–16)^****^
14-d smoothed	High	0.63%, 2.9 (1.68–5.1)^****^	0.71%, 4.2 (2.3–7.6)^****^	0.69%, 4.1 (2.3–7.4)^****^	0.65%, 3.8 (2.1–7.0)^****^
	Moderate-high	0.46%, 2.2 (1.22–3.8)^***^	0.25%, 1.49 (0.75–3.0)	0.30%, 1.79 (0.93–3.5)^**^	0.43%, 2.5 (1.35–4.8)^***^
	Moderate-low	0.26%, 1.22 (0.65–2.3)	0.41%, 2.4 (1.31–4.5)^***^	0.39%, 2.3 (1.22–4.3)^***^	0.32%, 1.84 (0.95–3.6)^**^
	Low	0.21%, Reference	0.17%, Reference	0.17%, Reference	0.17%, Reference
	High + RLI	0.99%, 7.1 (3.1–16)^****^	1.06%, 9.8 (4.3–23)^****^	1.03%, 9.7 (4.2–22)^****^	0.99%, 9.2 (3.9–22)^****^
28-d smoothed	High	0.51%, 2.2 (1.26–3.8)^***^	0.57%, 3.4 (1.81–6.2)^****^	0.57%, 3.4 (1.80–6.3)^****^	0.68%, 3.5 (1.99–6.3)^****^
	Moderate-high	0.44%, 1.87 (1.07–3.3)^**^	0.44%, 2.6 (1.37–4.9)^***^	0.49%, 2.9 (1.55–5.5)^***^	0.31%, 1.58 (0.83–3.01)
	Moderate-low	0.35%, 1.51 (0.86–2.7)	0.39%, 2.3 (1.22–4.4)^***^	0.34%, 2.0 (1.05–3.9)^**^	0.39%, 2.0 (1.10–3.7)^**^
	Low	0.23%, Reference	0.17%, Reference	0.17%, Reference	0.19%, Reference
	High + RLI	0.78%, 5.1 (2.2–11)^****^	0.86%, 8.2 (3.5–19.7)^****^	0.87%, 8.4 (3.5–20)^****^	1.04%, 8.5 (3.7–20)^****^
Rolling average ACWR	High	0.54%, 1.81 (1.12–2.9)^***^	0.56%, 2.5 (1.50–4.3)^****^	0.56%, 2.8 (1.48–3.9)^****^	0.46%, 1.30 (0.81–2.1)
	Moderate-high	0.30%, Reference	0.22%, Reference	0.20%, Reference	0.36%, Reference
	Moderate-low	0.29%, 0.96 (0.57–1.67)	0.38%, 1.72 (0.98–3.0)^**^	0.44%, 2.3 (1.27–4.0)^***^	0.35%, 0.98 (0.58–1.64)
	Low	0.30%, 1.01 (0.59–1.75)	0.23%, 1.02 (0.54–1.92)	0.22%, 1.14 (0.60–2.2)	0.22%, 0.62 (0.35–1.10)
	High + RLI	0.77%, 3.7 (1.78–7.8)^****^	0.82%, 5.4 (2.5–12)^****^	0.79%, 5.8 (2.7–12.5)^****^	0.70%, 2.8 (1.36–5.9)^***^
Smoothed ACWR	High	0.70%, 3.6 (2.1–6.1)^****^	0.83%, 8.0 (3.9–17)^****^	0.83%, 8.1 (3.9–17)^****^	0.79%, 5.1 (2.8–9.4)^****^
	Moderate-high	0.19%, Reference	0.10%, Reference	0.10%, Reference	0.16%, Reference
	Moderate-low	0.25%, 1.27 (0.68–2.3)	0.25%, 2.4 (1.06–2.6)^**^	0.28%, 2.7 (1.19–6.3)^***^	0.32%, 2.1 (1.04–4.0)^**^
	Low	0.34%, 1.75 (0.99–3.1)^**^	0.35%, 3.3 (1.54–7.7)^***^	0.32%, 3.1 (1.41–6.7)^***^	0.26%, 1.67 (0.82–3.3)
	High + RLI	0.93%, 6.8 (3.2–14)^****^	1.13%, 16 (6.4–40)^****^	1.12%, 16 (6.4–40)^****^	1.09%, 10 (4.6–23)^****^
Monotony	High	0.55%, 1.63 (1.00–2.7)^**^	0.61%, 2.5 (1.48–4.1)^***^	0.60%, 3.1 (1.81–5.3)^****^	0.67%, 2.6 (1.56–4.2)^****^
	Moderate-high	0.36%, 1.06 (0.63–1.80)	0.31%, 1.24 (0.72–2.14)	0.29%, 1.48 (0.82–2.7)	0.23%, 0.86 (0.48–1.57)
	Moderate-low	0.28%, 0.84 (0.48–1.47)	0.30%, 1.21 (0.72–2.04)	0.39%, 1.98 (1.18–3.4)^***^	0.31%, 1.17 (0.69–1.98)
	Low	0.34%, Reference	0.25%, Reference	0.19%, Reference	0.26%, Reference
	High + RLI	0.81%, 3.6 (1.69–7.5)^***^	0.91%, 5.4 (2.4–12.1)^****^	0.90%, 6.9 (3.0–16)^****^	1.02%, 5.7 (2.6–12)^****^
Strain	High	0.61%, 1.91 (1.18–3.1)^***^	0.58%, 2.1 (1.28–3.6)^***^	0.50%, 1.69 (1.01–2.8)^**^	0.59%, 2.2 (1.29–3.6)^***^
	Moderate-high	0.36%, 1.13 (0.66–1.92)	0.36%, 1.31 (0.75–2.3)	0.41%, 1.38 (0.81–2.4)	0.33%, 1.22 (0.69–2.2)
	Moderate-low	0.24%, 0.76 (0.42–1.36)	0.30%, 1.09 (0.61–1.95)	0.30%, 1.00 (0.56–1.76)	0.33%, 1.20 (0.68–2.1)
	Low	0.32%, Reference	0.27%, Reference	0.30%, Reference	0.27%, Reference
	High + RLI	0.92%, 4.3 (2.0–9.1)^****^	0.89%, 4.9 (2.2–11)^****^	0.77%,3.9 (1.77–8.5)^****^	0.91%, 5.0 (2.3–11)^****^

a *Values are injury hazard (risk per player per exposure day), hazard ratio (with 90% confidence limits). Substantial effects are in bold*.

Likelihood of increased risk of injuries: * possibly,

** *likely*,

*** *very likely*,

**** *most likely. RLI, recent leg injury; sRPE, session rating of perceived exertion; ACWR, acute-to-chronic workload ratio*.

Substantial individual differences in injury risk existed even after accounting for training load and history of previous leg injuries. For example, injury-prone players were at a more than five times higher risk of injuries compared to the robust players after adjusting for a 14-d smoothed Player Load and previous leg injuries (hazard ratio 5.4, 90% confidence limits 3.6–12). Similar differences of more than five times were observed after adjusting for other derived measures of training load.

The effects of high-risk levels for professional experience, sit-and-reach test, adductor squeeze tests, and mood after adjusting for training load and history of previous leg injuries are summarized in Table 6. Negligible differences in the hazard ratio of <5% were observed between the individual and adjusted effects for these high-risk levels. The combined effects of each of these high-risk levels with high training load and a recent leg injury are also shown in Table 6. Extremely large increases in the risk of injuries were observed when multiple risk factors were combined.

**Table 6:** The adjusted and combined effects of professional experience, musculoskeletal screening, and subjective wellness on the risk of injuries derived from part three of the analysis^a^.

**Selected variable**	**Level**	**Effect adjusted for acute load and history of previous leg injuries**	**Combined effect^b^ as compared to the lowest risk scenario**	**Combined effect^b^ as compared to the regular scenario**
Professional experience	7+ years	0.52%, 1.92 (1.04–3.6)^**^	1.64%, 15 (5.5–40)^****^	1.64%, 7.7 (3.5–17)^****^
Sit-and-reach test	Z-score < −1.5	0.49%, 2.5 (1.40–4.4)^***^	2.11%, 24 (8.2–68)^****^	2.11%, 17 (7.4–40)^****^
Adductor squeeze test 0	Z-score < −1.5	0.41%, 1.98 (1.03–3.8)^**^	1.77%, 19 (6.2–57)^****^	1.77%, 14 (5.6–35)^****^
Adductor squeeze test 45	Z-score < −1.5	0.35%, 1.66 (0.86–3.2)	1.52%, 16 (5.3–49)^****^	1.52%, 12 (4.7–29)^****^
Adductor squeeze test 90	Z-score < −1.5	0.51%, 2.9 (1.72–4.9)^****^	2.36%, 31 (11–89)^****^	2.36%, 22 (9.7–52)^****^
Mood	Z-score < −1.5	0.65%, 1.99 (1.08–3.7)^**^	2.12%, 15 (5.3–41)^****^	2.12%, 9.5 (4.0–23)^****^

a *Values are injury hazard (risk per player per exposure day), hazard ratio (with 90% confidence limits). Substantial effects are in bold*.

b Combined with high acute load and a recent leg injury.Likelihood of increased risk of injuries: *possibly,

** *likely*,

*** *very likely*,

**** *most likely*.

Substantial proportions of the effects of high ACWR were explained by acute load and previous leg injuries. The effect of high sRPE rolling average ACWR decreased by 28% from hazard ratio of 2.1 (90% confidence limits 1.32–3.3) to 1.50 (0.90–2.52) after adjusting for acute load (sRPE 7-d rolling average) and previous leg injuries. Similarly, the effect of high sRPE smoothed ACWR decreased by 38% from 4.0 (2.4–6.7) to 2.5 (1.4–4.5) after adjusting for acute load (sRPE 7-d smoothed) and previous leg injuries.

## Discussion

High absolute and relative measures of internal and external training load were associated with increased risk of injuries, and the magnitudes of the effects were considerably influenced by history of previous injuries. The effects for training load measures derived using exponentially weighted moving averages were typically larger than the effects for similar measures derived using rolling averages. A substantial proportion of the effects of high ACWR was explained by acute load and history of previous injuries. Having 7+ years of professional experience and substantial reductions in sit-and-reach test, adductor squeeze tests, and mood were also associated with a higher risk of injuries, and the effects of these risk factors were not confounded by training load and history of previous injuries. Combinations of multiple risk factors were associated with extremely large increases in the risk of injuries.

### Training load

Training induces physiological and biomechanical stress on physiological systems and body tissues. Accumulation of the negative effects of such stressors through vigorous training and/or inadequate recovery results in a reduced stress-bearing capacity of the tissues and a higher chance of failure of the adaptive mechanisms and injury (Kumar, 2001; Vanrenterghem et al., 2017). This process can explain the observed associations between high cumulative loads and increased risk of injuries, which is consistent with previous findings in various football codes (Rogalski et al., 2013; Cross et al., 2016; Malone et al., 2017). In the present study, the effects of high training load increased after adjusting for history of previous leg injuries. An injury episode results in low cumulative loads as the injured player goes through the rehabilitation and return to play process. A recently injured player is inherently at a higher risk of injuries upon return to play (Toohey et al., 2017), and as a result, the injury hazard for purely low training load tends to be overestimated. In other words, a proportion of the estimated injury hazard in the low training load level is due to the effects of recent injuries. In our study, accounting for history of previous leg injuries led to a lower estimate of injury hazard for low training load (which served as the reference level) and a higher injury hazard for high training load compared to the unadjusted (individual) values. These changes in turn translated into larger effects (hazard ratios) for high training load.

The only derived measures where adjusting for previous leg injuries resulted in smaller effects for the high levels were sRPE rolling average ACWR and sRPE smoothed ACWR. The effects were further reduced after we also adjusted for acute load. High acute load and previous injuries are known injury risk factors, which may contribute to a high ACWR, and we found that a substantial proportion of the effects of high ACWR on the increased risk of injuries is explained by these variables. Nevertheless, the associations between high ACWR and increased risk of injuries remained substantial even after adjusting for acute load and previous leg injuries, indicating that a high ACWR may represent a high-risk condition that is not identified by monitoring only acute load and previous injuries. Researchers and practitioners are advised to consider the reasons behind a high ACWR when interpreting their data. In addition, moderate-high levels of smoothed ACWR (as defined in Table 1) were associated with substantially lower risk of injuries compared to other levels even after adjusting for previous leg injuries. Similar protective effects have been observed for moderate-high rolling average ACWRs in rugby league and soccer (Hulin et al., 2016a; Malone et al., 2017).

In contrast to the sRPE-derived ACWRs, the effects of high GPS/accelerometer-derived ACWRs increased after adjusting for previous leg injuries. This unexpected finding is likely due to the fact that the majority of training for injured players during the rehabilitation period cannot be captured using the GPS/accelerometer units, resulting in inconsistent ACWRs at the early stages of return to full training. A number of studies have attempted to address this limitation by removing high ACWRs from the analysis when chronic loads were more than 1 SD or 2 SD below the mean, which limits the application of ACWR following an injury episode (Hulin et al., 2014, 2016b; Carey et al., 2017a). More research is required to better understand and address the issue of inconsistent ACWRs post-injury, especially for GPS/accelerometer-derived variables.

It should be noted that the training load of each day was included in calculation of the derived training load measures on that day. This step is important for practitioners and studies with a daily design, in view of the weekly periodization of training in elite settings. For example, high cumulative loads derived from previous days would not be a concern, when only a light recovery session is planned on a given day. Only seven of the 65 injuries in our study resulted in the injured player completing substantially lower training load than originally planned on the day of injury, which along with the daily analysis of data, minimized the risk of associating artificially low derived measures with injury incidences. The derived training loads in our study represent the actual workloads completed by players up to the moment of sustaining an injury. To apply this method in practice, an estimated training load of the upcoming day should be used to calculate the derived measures, in order to evaluate the load and injury risk of individual players, should they proceed to complete the training day as planned. The resulting information can then be used in the decision-making process in regards to training modification or team selection for individual players (Charlton et al., 2017). Several studies with weekly designs have similarly evaluated the effects of training load on injuries in the current week (Hulin et al., 2016b; Murray et al., 2017b; Windt et al., 2017a). The limited data availability did not allow us to split the derived training load measures into more than four levels for analysis. However, practitioners may further subdivide the training load levels to differentiate between high, very high, and extremely high derived training loads, which will provide additional practically relevant information. It is also possible that extreme values of some training load constructs are more sensitive than others in detecting high risk of injuries (Vanrenterghem et al., 2017).

### Exponentially weighted moving averages vs. rolling averages

This study is the first to have evaluated the effects of cumulative loads derived using both exponentially weighted moving averages and rolling averages on the risk of injuries. We demonstrated that cumulative load calculated using exponentially weighted moving averages is a better alternative to rolling average cumulative load in evaluating the risk of injuries. Similar conclusions can be made in regards to ACWRs. A recent study also found that exponentially weighted moving average ACWR (a relative measure of training load) was a more sensitive indicator of injury likelihood in Australian football than rolling average ACWR (Murray et al., 2017a). Exponentially weighted moving averages take into account the physiological principle that the effects of a training stimulus decay over time, while rolling averages assign the same level of importance to all observations in a time period (Hawley, 2002; Menaspà, 2017a). This emerging method of deriving training load has shown promising applications in evaluation of future match performance (Lazarus et al., 2017) and injury prevention (Murray et al., 2017a). In our study, the 14-d time period for cumulative smoothed loads (decay factor = 0.07) had the largest associations with the risk of injuries. Differences between sports in training loads, training periodization, and competition schedule (Gamble, 2006; Varley et al., 2014) may result in other decay factors to perform better under such different circumstances. Researchers are encouraged to explore with various decay factors to identify the one that works best in their sport and sample of athletes.

### History of previous injuries

History of previous injuries is a well-recognized injury risk factor and has been evaluated from two main perspectives. Recurrent injuries refer to the same injury type to the same site as the index (initial) injury, while subsequent injuries may or may not differ from the index injury in nature or location (Finch and Cook, 2014; Finch et al., 2017). In our study, we evaluated the effects of any previous injuries and previous leg injuries on subsequent soft tissue non-contact leg injuries, and found that recent injury history was associated with a substantial increase in the risk of injuries. History of previous leg injuries had a slightly larger effect on injury risk compared to the history of any previous injuries, as only soft tissue non-contact leg injuries were considered for analysis in the current study. We used the number of days since return to full training as a method of accounting for decaying effects of previous injuries.

Our results are in line with previous findings where recent injuries had a larger effect on injury recurrence compared to previous non-recent injuries (Orchard, 2001; Orchard et al., 2017). Complete resolution of deficits resulting from an injury episode may extend beyond the time of return to play (Orchard and Best, 2002; Verrall et al., 2006). Such deficits include reduced muscular strength and flexibility, proprioception, and general fitness, as well as altered running biomechanics and motor control (Mujika and Padilla, 2000; Dauty et al., 2003; Lee et al., 2009; Maniar et al., 2016), which collectively contribute to a higher risk of subsequent injuries in athletes with a history of a recent injury (Toohey et al., 2017).

There is a need for studies that make appropriate adjustments for potential confounders in evaluating athletes' injury risk profile (Toohey et al., 2017; Windt et al., 2017b). We found history of previous injuries to be a positive confounder (Meeuwisse, 1994b) for sRPE-derived ACWR and a negative confounder (Meeuwisse, 1994b) for other derived measures of training load. Studies investigating the effects of training load on injury risk should adjust for the decaying effects of previous injuries in order to obtain more accurate estimates.

### Professional experience

Players with 7+ years of professional experience were at a higher risk of injuries compared to the players with 3–6 years of professional experience. A number of AFL studies have reported similar findings, where more experienced players had a higher risk of injuries compared to the less experienced players (Gabbe et al., 2006; Rogalski et al., 2013; Colby et al., 2017b). The body's adaptive capacity in response to a training stimulus as well as the ability to recover from fatigue are thought to diminish as professional experience and therefore age increase (Maffey and Emery, 2007); however, little quality evidence specific to athletes exists to support these plausible speculations.

History of previous injuries has been identified as a confounder for age, as older players have likely sustained more injuries over their career, which may have predisposed them to subsequent injuries (Arnason et al., 2004). In our study, the effects of professional experience did not change substantially after adjusting for training load and history of previous injuries, possibly due to the fact that our records of history of previous injuries extended only as far as the season prior to the study period rather than the entire professional career of individual players. Our results indicate that the effects of professional experience on injury risk are independent from the effects of training load and history of injuries over the current and previous season. Professional experience remains an important factor to consider in relation to load management and injury prevention in professional athletes.

### Musculoskeletal screening

Substantial reductions in the sit-and-reach and adductor squeeze test scores were associated with an increased risk of injuries. The effects of musculoskeletal screening scores on the risk of injuries have been evaluated mostly in the form of pre-season screening. A limitation of this approach is that pre-season test scores reflect the condition of athletes only at that particular time (Whiteley, 2016). Musculoskeletal screening scores of elite Australian footballers have shown substantial week-to-week variations throughout the season (Esmaeili et al., 2018). Variation in the scores obtained from regular musculoskeletal screening, rather than the absolute values, may better reflect the condition of athletes, their response to prescribed training, and subsequently the risk of injuries (Paul et al., 2014; Esmaeili et al., 2017; Thorpe et al., 2017). Our findings provide evidence for these previous speculations on application of weekly musculoskeletal screening for injury prevention purposes. Larger reductions in the test scores (>1.5 SD vs. >1 SD) had slightly larger effects on the risk of injuries. Practitioners may use both these thresholds in a multi-level flagging system.

One study to date has evaluated the effects of substantial reductions (>1 SD) in weekly musculoskeletal screening scores on the risk of injuries in elite Australian footballers, and did not find substantial associations between these variables (Colby et al., 2017b). In this study, rolling season-to-date standard deviations were used to determine substantial reductions in the test scores, while in our study we used the pool of scores at each season phase of each season for this purpose. Creating z-scores using the rolling season-to-date approach may result in inconsistent scores at early stages of the season (Robertson et al., 2017). This methodological dissimilarity as well as possible differences in the implemented interventions in response to the screening scores can explain the difference in findings between the two studies.

The effects of reductions in screening scores did not substantially change after adjusting for the effects of previous leg injuries and training load. The screening tests in our study were conducted immediately prior to the first field training session of the week, which was planned on day 2 or 3 after a match (or main training session during pre-season). Changes in screening scores following matches generally return back to baseline within 2 days (Dawson et al., 2005; McLellan et al., 2011; Wollin et al., 2017). We have previously found that screening tests are not sensitive to training load (Esmaeili et al., 2018), which can explain the similarity between the unadjusted and adjusted effects of reductions in screening scores. Training load and previous leg injuries did not confound the effects of reductions in screening scores.

### Subjective wellness

Substantial reductions in mood were associated with increased risk of injuries, while no substantial effects were observed for similar reductions in the scores (feeling worse) for perceived fatigue, sleep quality, and stress. In addition, worse scores for general muscle soreness were associated with a lower risk of injuries. The likely reason behind such inconsistencies is the combination of changes in wellness scores in a weekly cycle and the daily analysis design used in our study. Wellness scores are generally at their lowest at early stages following a match (or the main training session during pre-season), and gradually return back to baseline before the next match (Thorpe et al., 2016; Gallo et al., 2017). The wellness z-scores in our study were created from the pool of the data for each item at each season phase for individual players. As a result, generally lower scores obtained earlier in a week coincided with light recovery sessions that inherently have a lower risk of injuries. On the other hand, typically higher wellness scores could have been recorded later in the week and before matches or main training sessions, which carry a higher risk of injuries. Future studies with larger sample sizes should evaluate the effects of changes in wellness scores on the risk of injuries while comparing the wellness scores on a given day to previous scores obtained on similar days of the weekly cycle (e.g., creating z-scores of each day post-match separately). It is also possible that the implemented interventions in response to the wellness scores at this elite environment mitigated the risk of injuries and contributed to the observed inconsistencies.

### The combined effects and decision-making

The multivariate and complex nature of sports injuries has repeatedly been emphasized in the literature (Meeuwisse, 1994a; Bahr and Holme, 2003; Bittencourt et al., 2016). Our findings indicate that combinations of multiple risk factors result in extremely large increases in the risk of injuries. One study with a weekly design recently evaluated the effects of multiple variables similar to the ones investigated in the current study on the risk of injuries (Colby et al., 2017b). The authors found that the predictive accuracy of the multivariate model (as measured via the area under curve) was substantially better than all the univariate models when tested against data that was used to develop the model (in-sample data). While history of previous injuries was not evaluated in this study, interactions between low chronic workloads and very high rolling average ACWRs were associated with increased risk of injuries in the subsequent week. Our results are in agreement with these findings, when we consider that a key contributor to low chronic workloads is recent injuries.

An important issue to consider in the decision-making process for injury prevention in an elite sports setting is the cost-benefit analysis of training and match participation for individual players (Gabbett et al., 2016). To better apply this concept, the absolute injury hazards associated with individual and combined risk factors should be considered in addition to the hazard ratios, along with the concept of acceptable injury risk (Orchard et al., 2005; Creighton et al., 2010; Charlton et al., 2017). In a hypothetical scenario based on our findings in regards to the injury hazards (as provided in the tables for a given day), a player with high acute cumulative load (as defined in this study and not extremely high), will have a < 5% chance of sustaining a soft tissue non-contact injury over seven exposure days if he remains in this high acute load level during this period. This scenario may not warrant an aggressive training modification, considering the low absolute risk of injury and the likely benefits of training and match participation over this period. It should also be noted that an injury risk of between 1.5–2% remains for other levels of acute load for the same period, which can be considered as the inherent risk of participating in physical activity at elite level. In a second scenario, the high acute cumulative load is accompanied by a recent leg injury and substantial reductions in the musculoskeletal screening scores. The injury risk now increases to over 15% over the next seven exposure days, if the athlete remains in the high-risk level for these risk factors. These examples highlight the importance of simultaneous consideration of multiple risk factors. We also demonstrated that injury-prone players are at a more than five times higher risk of injuries compared to robust players after accounting for training load and previous injuries. Consequently, in the second scenario the actual risk for the same period would be more than 25% for an injury-prone player and < 5% for a robust player. As evident from these hypothetical scenarios, and in agreement with previous recommendations (Charlton et al., 2017; Gabbett et al., 2017), the numbers stemming from athlete monitoring practices cannot replace the medical and conditioning staffs' expertise and knowledge of players. Prediction of injuries as a binary outcome (yes/no) has not yet been shown to possess the required accuracy, especially when tested against out-of-sample data (Carey et al., 2017b; Colby et al., 2017b; Fanchini et al., 2018). However, estimation of injury risk for individual players on a given day as a probability may assist practitioners with making better informed decisions in the quest for minimizing injury risk and maximizing athletic performance. Other factors potentially influencing such decisions include the stage of the season, the coach's philosophy, availability of quality substitute players, competition schedule, and quality of the opposition (Orchard et al., 2005; Creighton et al., 2010; Charlton et al., 2017). A combination of workloads, players' response to workloads, and other injury risk factors should be considered along with several decision modifiers to increase the chance of success for individual players and the team.

### Limitations

The current study is effectively a case study of a single Australian football team over two consecutive seasons. While the explained concepts behind our findings likely apply to other Australian football clubs and team sports in general, the exact thresholds for predictors and magnitudes of effects may differ in other environments. Australian football clubs are not allowed to track training loads during the off-season period and Christmas break, which could have negatively affected the precision of our estimates (Buchheit, 2017). The z-scores for musculoskeletal screening and subjective wellness were calculated using the pool of data at each season phase of each season, which is a rather retrospective approach. This approach was taken to avoid obtaining likely inconsistent scores in the absence of enough historical data at early stages of each season phase (Robertson et al., 2017). In a practical setting, historical data from the previous season may be used at early stages of each season to overcome this limitation. The ACWR measures in our study were calculated using the coupled approach and the 7–28 day time windows, while the use of uncoupled approach and other time windows could potentially be more appropriate (Carey et al., 2017a; Lolli et al., 2017; Windt and Gabbett, 2018). In our study, a rather large number of independent variables were evaluated and multiple comparisons were made, which automatically increase the risk of type I error (false positive). However, this limitation is not concerning here as the majority of the independent variables in our study (as opposed to only a small selection) showed substantial associations with injury risk.

## Conclusion

High absolute and relative measures of training load were associated with increased risk of injuries. These effects were positively or negatively confounded by history of previous injuries. History of a recent injury, long-term experience at professional level, and substantial reductions in a selection of musculoskeletal screening and subjective wellness scores were also associated with increased risk of injuries. Combinations of multiple risk factors resulted in extremely large increases in the risk of injuries. The information from athlete monitoring practices should be interpreted collectively and used as a part of the injury prevention/player management process along with consideration of individual differences in the risk of injuries.

## Author contributions

AE, RA, AS, WH, and GE conceived and designed the study; WH, AE, and BL analyzed the data; AE, WH, and RA interpreted the results; AE drafted the manuscript and prepared the tables; AE, RA, WH, BL, GE, and AS edited, critically revised the manuscript, and approved the final version.

### Conflict of interest statement

The authors declare that the research was conducted in the absence of any commercial or financial relationships that could be construed as a potential conflict of interest.
